# Supplementary material for: Molecular identification based on mtDNA analysis of commercial crustaceans in the coastal Amazon: exotic species, cryptic diversity, and implications for sustainable fisheries in northern Brazil
Source: PeerJ. 2025 Oct 13;13:e19586. doi: 10.7717/peerj.19586 (PMC12530202; doi:10.7717/peerj.19586)
Supplement: Supplemental Information 1 — The statistical support values are shown on internodes. [file peerj-13-19586-s001.pdf]

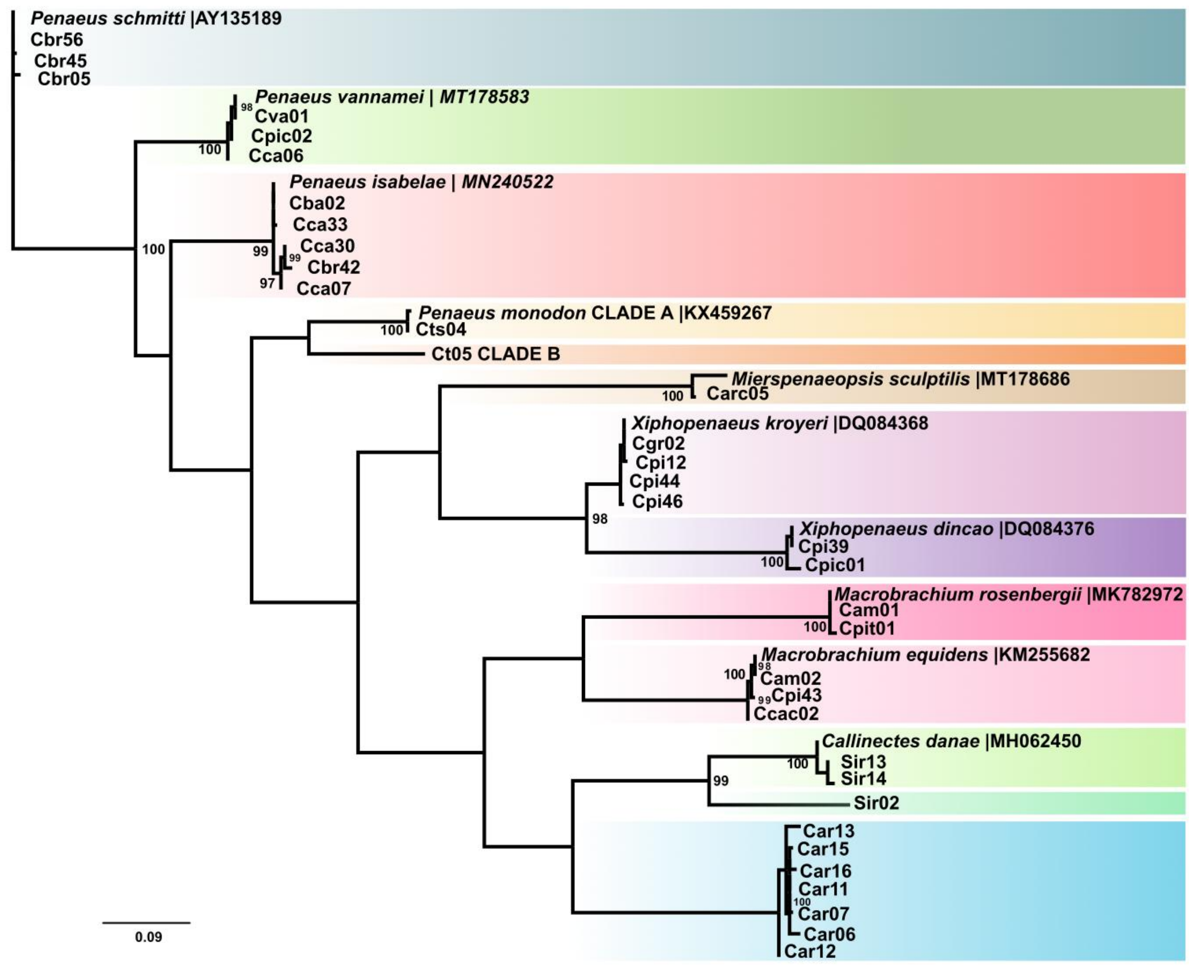

Fig. S1. Maximum likelihood (ML) tree showing the haplotypes of crustaceans based on the second region (region II) of the Cytochrome C Oxidase Subunit I (COI) gene, including the reference sequences from public databases. The statistical support values are shown on internodes.

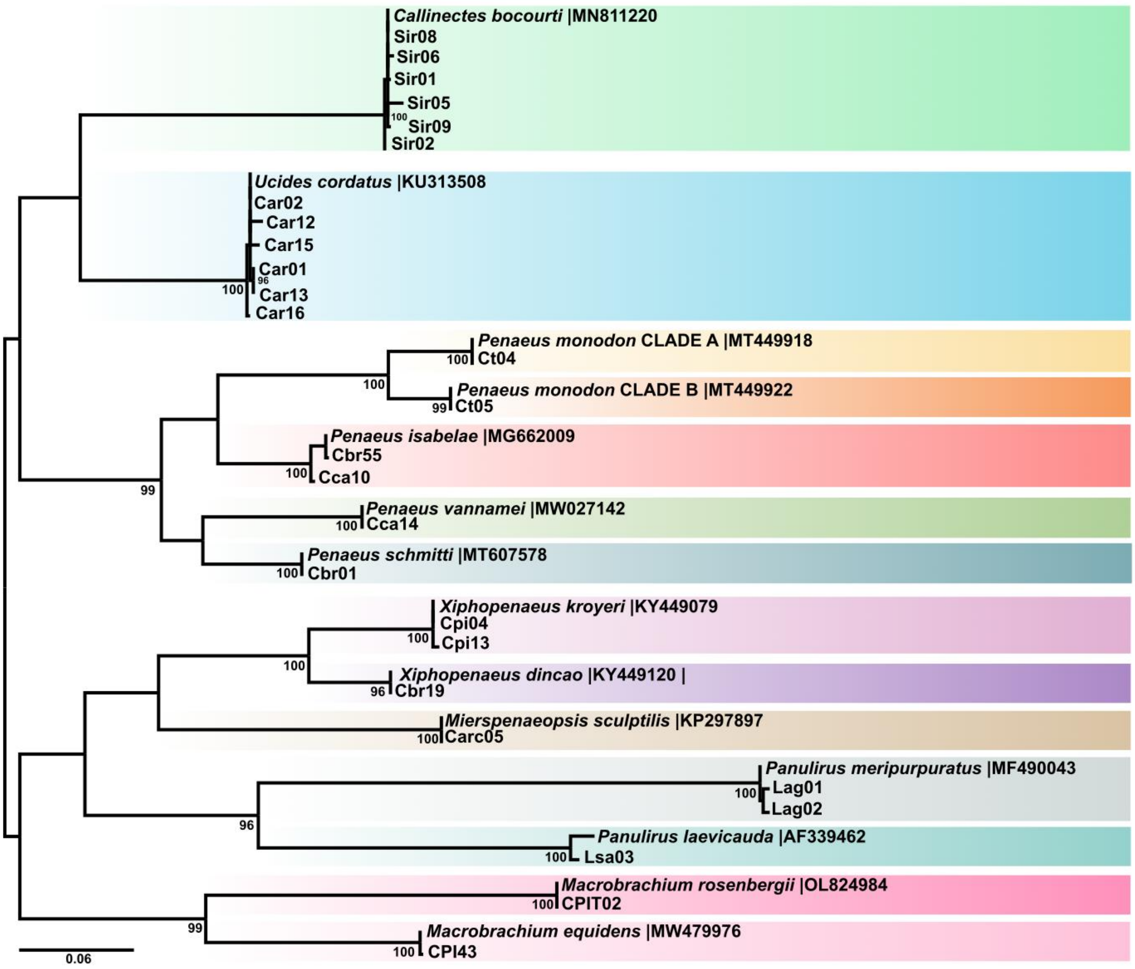

Fig. S2. Maximum likelihood (ML) tree showing the haplotypes of crustaceans based on the first region (barcode region or region I) of the Cytochrome C Oxidase Subunit I (COI) gene, including the reference sequences from public databases. The statistical support values are shown on internodes.
